# Urokinase plasminogen activator protects cardiac myocytes from oxidative damage and apoptosis via hOGG1 induction

**DOI:** 10.1007/s10495-017-1388-9

**Published:** 2017-06-22

**Authors:** Philipp J. Hohensinner, Nikol Takacs, Christoph Kaun, Barbara Thaler, Konstantin A. Krychtiuk, Stefan Pfaffenberger, Arezu Aliabadi, Andreas Zuckermann, Kurt Huber, Johann Wojta

**Affiliations:** 1 Department of Internal Medicine II, Cardiology, Medical University of Vienna, Währingergürtel 18-20, 1090 Vienna, Austria; 2 Department of Surgery, Medical University of Vienna, Vienna, Austria; 3 3rd Medical Department, Wilhelminenhospital, Vienna, Austria; 4 Ludwig Boltzmann Cluster for Cardiovascular Research, Vienna, Austria

**Keywords:** Urokinase plasminogen activator, Oxidative DNA damage, hOGG1, P53 pathway

## Abstract

The role of uPA in tissue remodeling and cell migration is already well established. In addition, uPA was reported to stabilize p53, a key cell cycle control, DNA repair and apoptosis initiation protein. We aimed to determine the role of uPA-uPAR signaling towards cell survival or apoptosis in human adult cardiac myocytes (HACM). HACM were stimulated with uPA and DNA damage was inflicted by incubating cells with 200 µM H_2_O_2_. To analyze for apoptotic cells we applied TUNEL staining. Oxidative damage foci were analyzed by staining for 8-oxoguanine base pairs. In vivo qPCR analysis from RNA extracted from failing human hearts demonstrated a close relation of uPA with apoptosis and the p53 pathway. Furthermore, we observed a close correlation of uPA and p53 protein in homogenized tissue lysates. In vitro studies revealed that uPA preincubation protected HACM from oxidative damage induced cell death and reduced oxidative damage foci. uPA protection is independent of its catalytic activity, as the amino terminal fragment of uPA showed similar protection. A key enzyme for repairing oxidative DNA damage is the p53 target hOGG1. We found a significant increase of hOGG1 after pretreatment of HACM with uPA. Knockdown of hOGG1 completely abrogated the protective effect of uPA. We conclude that uPA might have a tissue protective role in human hearts besides its role in tissue remodeling. Tissue protection is mediated by the DNA repair protein hOGG1. This might be beneficial during tissue remodeling and thus could be a target for therapeutic approaches in the diseased heart.

## Introduction

Urokinase-type plasminogen activator (uPA) is one of two serine proteases in the human plasminogen activation system [1]. Whereas tissue-type PA (tPA) through its fibrin specificity is mainly responsible for fibrin clot lysis, uPA bound to its cell surface receptor uPA receptor (uPAR) modulates extracellular proteolysis [2]. Thereby uPAR localizes uPA and subsequently the activation of plasminogen to plasmin to the cell surface thus giving the cell spatial control over degradation of extracellular components such as laminin and fibronectin [2, 3]. In addition, uPA can activate pro-matrixmetalloproteases to their active form and hence modulate a wide variety of extracellular matrix degrading enzymes. uPAR is widely expressed in various tissues including lungs, kidney, spleen, liver and heart [4]. A central role for uPA and uPAR was already described in different physiological and pathological events depending on extracellular proteolysis and tissue remodeling such as embryonic development, wound healing, tumor growth and inflammatory conditions [5–8]. In cancer high uPA and uPAR expression are markers for adverse outcome possibly due to increased cancer cell motility as well as to tissue degrading and angiogenesis enhancing properties [8–10]. In addition, uPA expression and function was shown to be crucial after myocardial infarction. uPA knockout mice were completely protected against cardiac rupture after myocardial infarction but suffered from impaired infarct revascularization suggesting a time dependent negative or beneficial effect of uPA availability [11]. uPA levels were increased in scar tissue compared to viable myocardium 3 months post myocardial infarction indicating ongoing uPA activation at sites of tissue remodeling and inflammation [12].

In addition to downstream signaling mainly associated with migration, uPA was reported to stabilize p53, a key enzyme in cell cycle control, DNA repair and apoptosis induction via beta1-integrin [13]. Primarily, p53 is a transcription factor involved in apoptosis signaling pathways by initiating transcription of proteins such as PUMA, Bax and Bid [14]. p53 can bind and inhibit Bcl-2 family members related to a direct apoptosis induction [15]. DNA damage repair enhancement is mainly considered to be regulated by p53 via orchestrating cell cycle arrest. Additionally, p53 directly regulates genes involved in nucleotide excision repair and base excision repair (BER) including the enzyme capable of removing 8-oxo-7,8-dihydroguanine (8-oxoG), the main oxidative DNA damage, termed hOGG1 [16–18].

Increased oxidative stress together with DNA damage and cytokine production activate apoptosis in the human heart. Apoptosis of cardiac myocytes can occur in patients suffering from myocardial infarction, dilated cardiomyopathy and end stage heart failure [19]. In addition to apoptosis, these conditions are characterized by enhanced matrix remodeling [20]. Although uPA has already been linked to heart tissue remodeling as well as to p53 and its downstream mediators, no data is available that addresses the question whether uPA might be actively involved in apoptosis or DNA repair induction in failing hearts. We therefore investigated, if uPA is associated with apoptosis in vivo and whether uPA might shift the balance within a cell towards cell death or DNA repair.

## Materials and methods

### Heart tissue

Heart tissue was obtained from explanted hearts of 21 patients undergoing heart transplantation and used for RNA preparation. From 10 of these samples we also obtained protein lysates. Paraffin embedded tissue sections were available from 11 samples. All tissue for RNA and protein analysis was taken from the left ventricle. Independently, myocardial tissue from six donors was used to prepare human adult cardiac myocytes. All human material was obtained and processed according to the recommendations of the hospital’s ethics committee and security board, including informed consent.

### Immunohistochemistry

Paraffin embedded human left ventricular heart tissue from explanted failing hearts from patients undergoing heart transplantation was stained for cleaved caspase 3 with a monoclonal rabbit-antibody (Cell Signaling, USA; 1:400) as reported previously [21]. Staining for 8-oxoG with a monoclonal mouse-antibody (Abcam, UK; 1:400) was performed as suggested by the manufacturer’s instructions. Fluorescence labeled secondary antibodies (R&D System, USA; 1:250) were applied overnight at 4 °C. DAPI (Sigma Aldrich, USA) was applied for nuclear staining and slides were embedded in ProLong Gold antifade (Life Technologies, USA). Mason Trichrome staining was performed using standard protocols (Sigma Aldrich, USA). Images were taken on a Zeiss Axiovision microscope (Carl Zeiss, Germany).

### RNA isolation and qPCR

RNA was isolated using high pure RNA tissue kit (Roche, Switzerland) as described in the manufacturer’s instructions. cDNA was generated from equal amounts of RNA per experiment using a Promega GoScript reverse transcription system (Promega, USA). Quantitative PCR (qPCR) was performed on a Roche Light Cycler 480 system (Roche, Switzerland) using the universal probe library (UPL) and the respective Roche kit. Primers were designed using the online UPL tool (BAX forward 5′atgttttctgacggcaacttc3′, reverse 5′atcagttccggcaccttg3′, UPL 57; GAPDH forward 5′agccacatcgctcagacac3′, reverse 5′gcccaatacgaccaaatcc3′, UPL 60; hOGG1 forward 5′ctgcatcctgcctggagt3′, reverse 5′gcctggggcttgtctagg3′, UPL 47; p53 forward 5′aggccttggaactcaaggat3′, reverse 5′ccctttttggacttcaggtg3′, UPL 12; uPA forward 5′ttgctcaccacaacgacatt3′, reverse 5′ggcaggcagatggtctgtat3′, UPL 46; uPAR forward 5′gccttaccgaggttgtgtgt3′, reverse 5′cttcgggaataggtgacagc3′, UPL 37). PCR conditions consisted of an initial step of 10 min at 95 °C followed by 50 cycles of 95 °C for 15 s and 60 °C for 30 s.

### Protein determination

To evaluate protein levels of uPA and p53 in failing heart tissue we used a ball mill (Retsch, Germany) to homogenize the snap frozen tissue in phosphate buffered saline (PBS), pH 7.4. Specific ELISAs for uPA (R&D System) and p53 (Affymetrix, USA) were used according to the manufacturer’s instructions. Specific protein levels were normalized to total protein as measured by NanoDrop (Thermo Fisher, USA).

### Cell culture and stimulation

Human adult cardiac myocytes (HACM) were isolated, characterized and maintained as described previously [22, 23]. Cells were serum starved in serum free medium (SFM) containing M199 supplemented with 0.1% bovine serum albumin (BSA; Sigma Aldrich, USA) 24 h prior to experiments. HACM were either incubated with SFM, stimulated for 24 h with uPA (Sekisui Diagnostics, Germany; specific activity 11 ng/U) at 100 U/ml or uPA aminoterminal fragment (ATF; Sekisui Diagnostics) at 50 ng/ml. For oxidative stress induction cells were treated with 200 µM H_2_O_2_ (Sigma Aldrich) for 2 h. Apoptosis was determined 24 h after H_2_O_2_ treatment using TdT-mediated dUTP-biotin nick end labeling (TUNEL) staining.

### TUNEL staining

To visualize DNA fragmentation in HACM TUNEL staining was performed using the in situ cell death detection kit (Roche) according to the manufacturer’s instructions. Apoptotic cells are stained in green. Images were visualized on a Zeiss Axiovision microscope using a 40× objective.

### 8-oxoguanine staining

8-oxoguanine was stained as published recently [24]. In short, HACM were fixed in 3.5% formaldehyde (Sigma Aldrich) and washed three times in PBS. After Triton X100 (Sigma Aldrich) permeabilization, samples were blocked for 1 h with 2% BSA in PBS. Mouse monoclonal anti 8-oxoguanine antibody (Abcam, UK; 1:200) was applied for 2 h at room temperature followed by secondary donkey anti mouse antibody (R&D; 1:200) at 4 °C overnight. Images were visualized on a Zeiss Axiovision microscope using a 40× objective and foci were counted in at least ten nuclei per condition manually.

### hOGG1 quantification

HACM were trypsinized, fixed in 1.5% formaldehyde (Sigma Aldrich) and permeabilized with methanol (Sigma Aldrich). Afterwards, cells were washed with PBS containing 1% BSA and stained with a polyclonal goat anti-hOGG1 antibody (Santa Cruz, USA; 1:200) and labeled with a FITC conjugated secondary donkey anti goat antibody (Santa Cruz; 1:200). Cells were analyzed on a FACS Canto II using FACS Diva software.

### si-RNA mediated knock down and hOGG1 overexpression

HACM were transfected with small interference RNA (si-RNA) for hOGG1 or with control siRNA (Dharmacon smart pool, USA), both at 100 nM, by electroporation at 200 V and 1200 µF with a Gene Pulser Xcell system (BioRad, USA). The same electroporation conditions were used for overexpression of hOGG1 in cardiac myocytes. hOGG1 plasmid was a gift from David Sidransky (Johns Hopkins University, Baltimore; plasmid provided by Addgene) [25].

### Statistics

All data is presented as mean ± SD with the number of experiments n given in the figure legend. Differences between two groups were tested using two-tailed Student’s *t*-test. A p-value of p ≤ 0.05 was considered statistically significant. Pearson correlation was calculated using SPSS (IBM, USA), Pearson correlation coefficient is given as r, p ≤ 0.05 was considered significant.

## Results

**Fig. 1:**
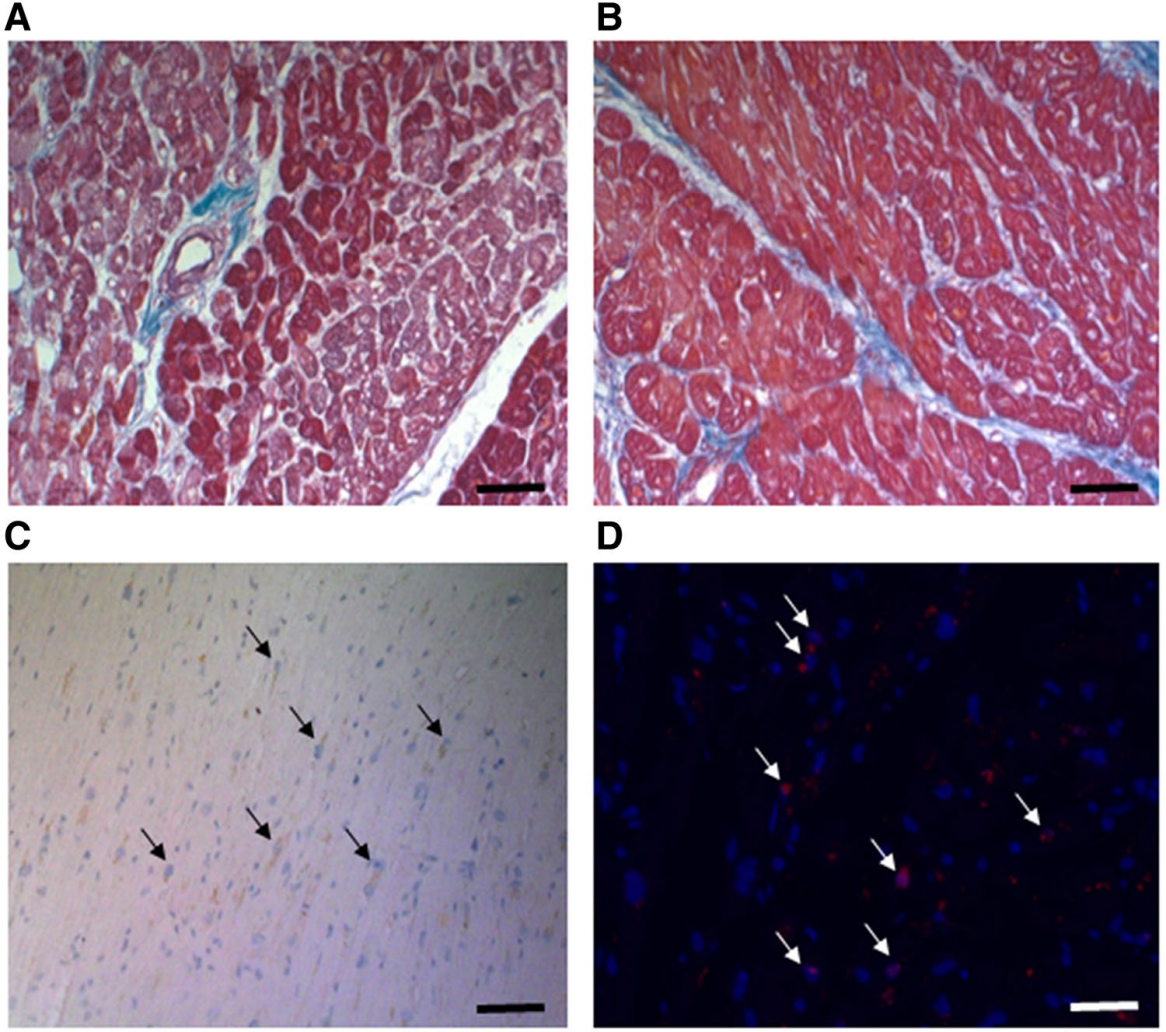
Tissue remodeling, apoptosis and oxidative stress in failing human heart sections. Human heart sections were stained by Mason Trichrome (**A** & **B**) to analyze collagen deposition (*blue*) in muscle sections (*red*). Apoptotic cells were identified by staining for cleaved caspase 3 (**C**) as indicated in “Materials and methods”, nuclei were counterstained using hematoxylin. Finally, oxidative damage was visualized by staining for 8oxoG lesions (**D**) by fluorescence immunohistochemistry (*red*) with DAPI counterstained nuclei (*blue*). *Black bars* represent 100 µm, *white bar* 50 µm

Failing hearts are characterized by changes in extracellular matrix, apoptotic cells including cardiac myocytes and cells damaged by oxidative stress. Mason trichrome stainings of heart tissue (n = 11) revealed large areas of collagen around vessels (Fig. 1A) and within muscle tissue (Fig. 1B). However, heart tissues from different donors were affected differently by the extent of collagen deposition ranging from 2% of the microscopic view to 13%. Similar to extracellular matrix deposition, all heart tissue sections contained apoptotic cells identified as cleaved caspase 3 positive cells (Fig. 1C). Of note, there was neither a correlation of apoptotic cells with the amount of extracellular matrix deposition nor was there a difference between ischaemic (n = 5) and dilatative cardiomyopathy (n = 6) for collagen deposition or cleaved caspase 3 positive cells in our small cohort. In addition, all failing heart tissue sections stained positive for oxidative DNA damage as indicated by nuclei staining positive for 8-oxoG lesions (Fig. 1D). There was no difference in oxidative damage between dilatative and ischaemic cardiomyopathy. 8-oxoG positive cells did not correlate with matrix deposition, but correlated strongly with caspase-3 positive cells (r = 0.607, p = 0.05).

**Fig. 2:**
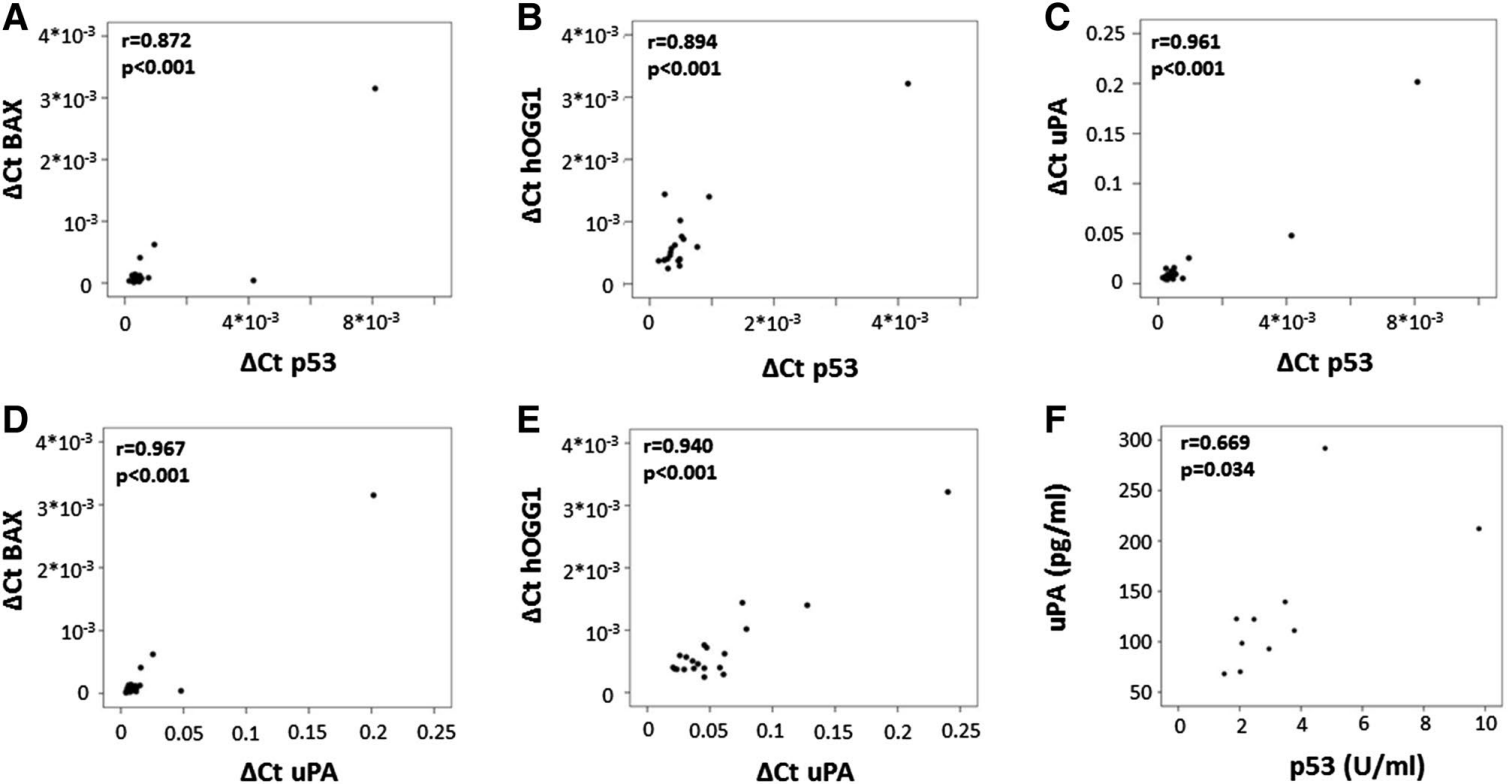
mRNA correlations in explanted left ventricular human heart tissue. RNA was isolated from human heart tissue and mRNA levels for p53, BAX, hOGG1 and uPA were determined, normalized to GAPDH, and correlated as indicated in “Materials and methods”. Values for the respective mRNA are given in ΔCt (**A**–**E**). In addition, uPA and p53 protein was determined by respective ELISA and normalized to total protein input as indicated in “Materials and methods” (**F**)

To determine, if the p53 pathway is activated in human hearts, we isolated RNA from left ventricular tissue of failing hearts from 21 patients. All of the samples expressed p53. In addition, p53 mRNA levels correlated with the expression of two p53 target genes, namely the proapoptotic BAX protein (r = 0.872, p < 0.001, Fig. 2A) and the DNA damage repair protein hOGG1 (r = 0.894, p < 0.001, Fig. 2B). Of note, one heart tissue sample did not give a positive signal for hOGG1. In addition, we determined mRNA expression levels of uPA and investigated if they correlated with the expression levels of p53. We found a strong correlation of uPA mRNA levels with mRNA levels of p53 (r = 0.961, p < 0.001, Fig. 2C) and with downstream targets of p53, BAX (r = 0.967, p < 0.001, Fig. 2D) and hOGG1 (r = 0.940, p < 0.001, Fig. 2E). To evaluate, if mRNA correlations would be reflected also on protein level we analyzed the correlation of p53 protein and uPA protein in lysates from failing human heart tissues (n = 10). Similarly to the mRNA data, p53 and uPA protein levels correlated significantly (r = 0.669, p = 0.03; Fig. 2F).

**Fig. 3:**
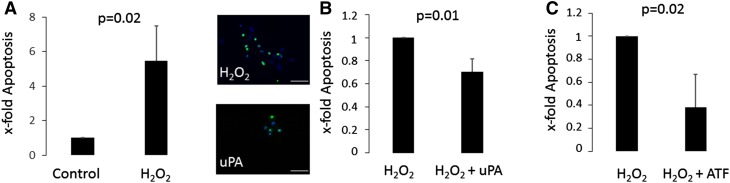
Human adult cardiac myocytes are protected from H_2_O_2_-induced apoptosis by uPA and ATF. Human adult cardiac myocytes (HACM) were incubated with 200 µM H_2_O_2_ or left untreated for 24 h (**A**). HACM were treated with 100 U/ml uPA (**B**) or 50 ng/ml ATF (**C**) or left untreated for 24 h before addition of 200 µM H_2_O_2_ for 24 h. Apoptosis was quantified as described in “Materials and methods”, apoptotic cell nuclei appear as* green*. Values are given as x-fold apoptotic cells over respective control and represent mean of 3 determinations ± SD of five representative microscopy fields each. The *inset* to **B** shows representative TUNEL staining images of the different conditions, the *white bar* represents 60 µm

Our data indicates that uPA in failing human hearts is associated with the occurrence of apoptosis and with the activation of the p53 pathway. To evaluate if these correlations are causative, we analyzed the effect of uPA on apoptosis in resting and oxidatively stressed human cardiac myocytes in vitro. uPA treatment without oxidative stress did not alter apoptotic numbers in cardiac myocytes (data not shown). Upon treatment with 200 µM H_2_O_2_ apoptotic rates of cardiac myocytes increased almost six fold (Fig. 3A). When cardiomyocytes were pretreated with uPA prior to oxidative stress induction, apoptotic rates were reduced by 0.3 ± 0.1 fold compared to cells not receiving uPA (p = 0.01, Fig. 3B). Of note, uPA stimulation slightly but significantly increased uPAR mRNA expression in cardiac myocytes (151 ± 27%, p = 0.03 after uPA stimulation). Full length uPA is a proteolytic active protein. However, intracellular signaling upon binding to its receptor does not require proteolytic activity [2]. To evaluate, if proteolytic activity is necessary to protect cells from oxidative damage induced cell death we used the aminoterminal fragment (ATF) of uPA. The ATF is devoid of the proteolytic domain but able to bind to uPAR and activate intracellular signaling [13]. Our results indicated that pretreatment of HACM with the ATF alone was similarly effective in protecting cells from oxidative injury induced cell apoptosis as pretreatment of the cells with full length uPA (Fig. 3C).

**Fig. 4:**
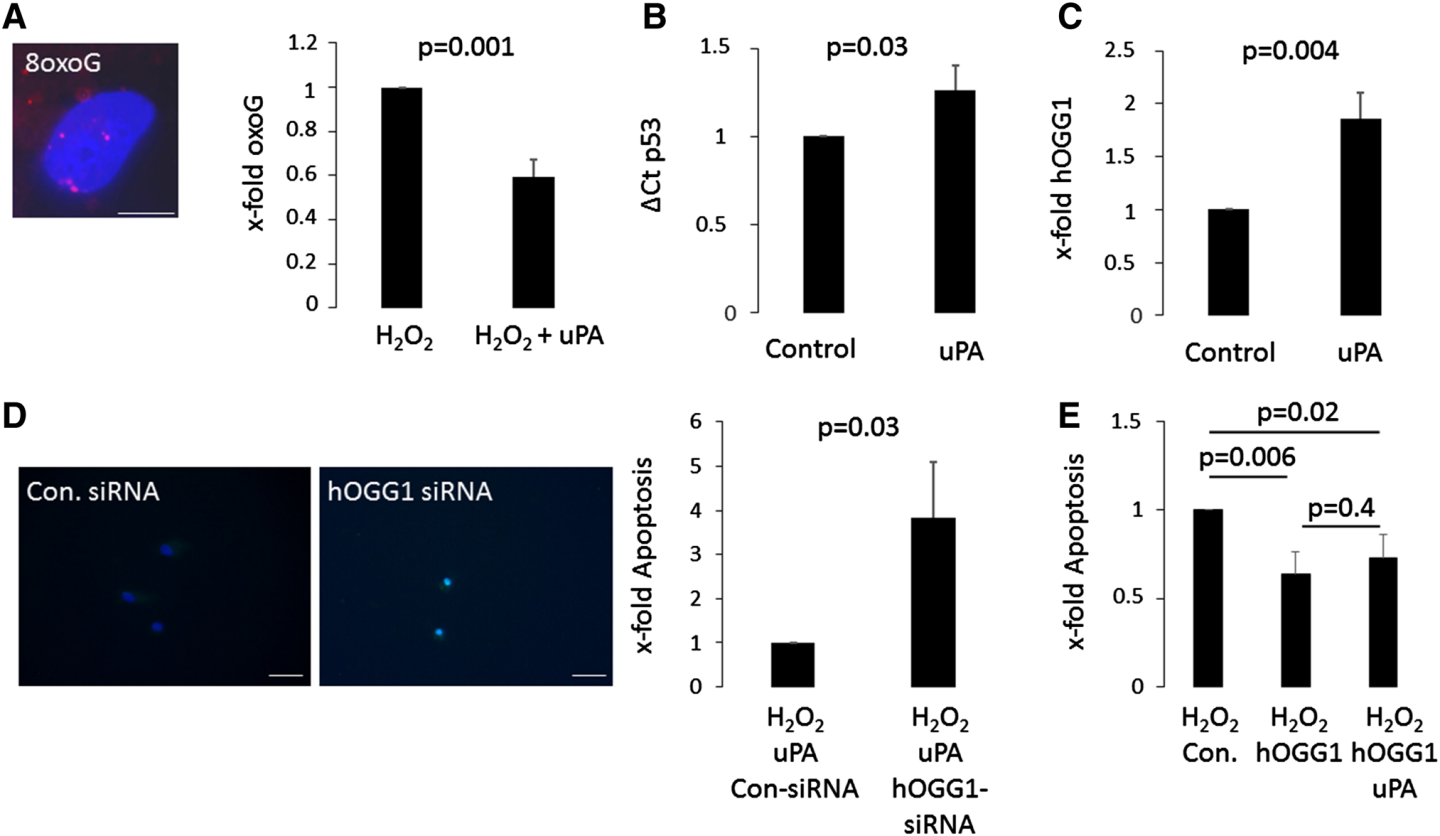
uPA protects human adult cardiac myocytes via hOGG1. Human adult cardiac myocytes (HACM), pretreated with 100 U/ml uPA for 24 h or left untreated, were incubated with 200 µM H_2_O_2_ for 2 h and 8-oxoguanine (8oxoG) lesions in cellular nuclei were determined after 24 h by immunofluorescence staining as indicated in “Materials and methods”. Values are given as x-fold oxoG lesions and represent mean of 3 determinations ± SD. The* inset* shows a representative image of a nucleus with 8oxoG lesions in *red* (**A**, *bar* represents 20 µm). mRNA levels for p53 were determined by qPCR after 2 h and normalized to GAPDH (**B**) and levels for hOGG1 (**C**) were determined by flow cytometry after 24 h of uPA treatment as indicated in “Materials and methods”. Values are given as ΔCt for p53 (**B**) and x-fold mean fluorescent intensity of hOGG1 (**C**) over untreated controls and represent mean of 3 determinations ± SD. HACM, transfected with hOGG1-siRNA or with control-siRNA as described in “Materials and methods” and pretreated with 100 U/ml uPA for 24 h were incubated with 200 µM H_2_O_2_ for 2 h. Apoptosis was quantified as described in “Materials and methods”. Values are given as x-fold apoptotic cells over control and represent mean of 3 determinations ± SD. The *inset* shows representative TUNEL staining images of the different conditions, the *white bar* represents 60 µm (**D**). HACM overexpressing hOGG1 or a control vector (Con.) were incubated with 200 µM H_2_O_2_ for 2 h. Apoptosis was quantified as described in “Materials and methods”. Values are given as x-fold apoptotic cells over control and represent mean of 3 determinations ± SD (**E**)

Oxidation of guanine is the major oxidative damage occurring in DNA [26]. We therefore determined the effect of uPA pretreatment on the occurrence of 8-oxoG foci at the DNA. uPA pretreatment reduced oxoguanine foci by 0.4 ± 0.1 fold of H_2_O_2_ treated HACM (p = 0.001, Fig. 4A). In our in vivo data we found a close correlation of uPA expression levels with p53 mRNA levels and with mRNA levels of the p53 target hOGG1. mRNA levels for p53 were already increased in vitro after 2 h uPA treatment 1.3 fold compared to untreated controls (p = 0.03, Fig. 4B). hOGG1 is a base excision repair protein capable of removing 8-oxoG base pairs and hence ensuring DNA integrity [17]. We therefore analyzed, if uPA is capable of increasing hOGG1 protein levels in cardiac myocytes. After 24 h of uPA stimulation, cardiac myocytes showed a 1.9 fold increase in mean fluorescence intensity for hOGG1 as compared to control (p = 0.004; Fig. 4C). To determine, if hOGG1 upregulation is a substantial part in uPA induced protection we used an si-RNA based knockdown approach. HACM were electroporated to allow si-RNA entry and allowed to recover for 24 h. For the knockdown experiments we omitted the 24 h starvation period prior to uPA stimulation to guarantee persistent si-RNA mediated hOGG1 knock down. Successful knockdown of hOGG1 was monitored by qPCR (knockdown to 5 ± 7% of control, p < 0.001; data not shown). uPA did not have a protective effect after knockdown of hOGG1. Apoptotic cell nuclei increased 3.8 ± 1.2 fold (p = 0.03) in cardiac myocytes with a hOGG1 knockdown compared to control knockdown cells after H_2_O_2_ treatment (Fig. 4D). uPA might however induce a variety of downstream genes. To further characterize the role of hOGG1 in cardiac cells we overexpressed hOGG1 in cardiac myocytes. Cell culture conditions were equivalent to the conditions used for the si-RNA knock down. Successful transfection was monitored using qPCR. Upon overexpression of hOGG1 we found an overall decrease in apoptotic nuclei. Adding uPA pretreatment to hOGG1 overexpression did not have additional beneficial effects indicating the importance of uPA mediated hOGG1 protection (Fig. 4E).

## Discussion

uPA is a serine protease mainly associated with tissue remodeling and cell migration [2]. In addition, we report in our current study a correlation of mRNA levels of p53 and its downstream targets with uPA mRNA levels, indicating a spatial connection of uPA availability and DNA damage. As p53 and its downstream mediators possess both the ability to induce apoptosis and protect cells with DNA damage we investigated in an in vitro approach the molecular consequence of uPA availability on cell fate decisions. Our data indicate that uPA stimulation induces the DNA repair protein hOGG1. The increased DNA repair capability seems to be the main mode of action of apoptosis protection of uPA.

uPA and uPAR have already been associated with cell survival in cancer cells. Inhibition of uPAR induced apotosis in melanoma cells and breast cancer cells [27, 28]. In addition, downregulation of uPAR and uPA was reported to activate caspase mediated apoptosis [29]. High levels of mRNA for uPA were shown to be connected with TRAIL responsive apoptosis in cancer cells. Depletion of uPA mRNA or uPAR lead to increased cell death possibly via reduced ERK1/2 prosurvival signaling upon deletion [30]. However, changes in p53 pathway targets were not analyzed. To further underpin the possible role of uPA in apoptotic fate determination, uPA was found to induce survival or proapoptotic proteins depending on the used stimulus in human mesangial cells [31]. In addition, in endothelial cells uPA was reported to transcriptionally activate X-linked inhibitor of apoptosis protein again leading to apoptosis inhibition [32]. Together, the current data available describes a possible cell type specific prosurvival property of uPA. We add to the current knowledge a novel protective pathway in adult cardiac myocytes. Our data strongly indicates that the BER protein hOGG1 is induced by uPA stimulation, and protects cardiac myocytes from oxidative damage induced apoptosis. Upon overexpression of hOGG1 we did not observe additional beneficial effects of uPA stimulation. The protective effect of hOGG1 is mediated by a reduced oxidative burden on DNA with increased repair of 8-oxoG base pairs as indicated by reduced 8-oxoG foci.

Several pathways have been already associated with hOGG1 induction. In general, the hOGG1 promoter does not contain TATA or CAAT boxes, suggesting that hOGG1 is consistently expressed [33]. Studies in a p53 knock out cell line have further pointed to p53 as an active regulator of hOGG1 expression as p53 binds to its putative cis-elements within the hOGG1 promoter and enhances transcription [18]. In addition, there seems to be a feedback loop between hOGG1 and p53 as hOGG1 protects cells against H_2_O_2_ induced apoptosis upstream of the p53 dependent apoptotic pathway [34]. Increased levels of hOGG1 could therefore be indicative of functional and ongoing oxidative stress repair and hence inhibit p53 induced apoptosis. We speculate that the massive increase of apoptosis in hOGG1 knockout cells might therefore not only be caused by oxidative stress but also by an increased p53 dependent apoptotic pathway.

In conclusion, we demonstrate a direct link between the serine protease uPA and the BER protein hOGG1 that is independent of the catalytic activity of uPA. In vitro experiments demonstrated the ability of uPA to induce hOGG1 and hence increase protection from oxidative DNA damage. We speculate that this link might be beneficial during tissue remodeling and angiogenesis, as the restoration of blood flow is usually accompanied by increased oxidative stress in the heart, and thus could be a target for therapeutic approaches to interfere with this process in the diseased heart.
